# Heart failure with improved ejection fraction: patient characteristics, clinical outcomes and predictors for improvement

**DOI:** 10.3389/fcvm.2024.1378955

**Published:** 2024-07-17

**Authors:** Amitai Segev, Benny Avrahamy, Alexander Fardman, Shlomi Matetzky, Dov Freimark, Ohad Regev, Rafael Kuperstein, Avishay Grupper

**Affiliations:** ^1^ Cardiovascular Division, Chaim Sheba Medical Center, Tel Hashomer, Ramat-Gan, Israel; ^2^ Faculty of Health Sciences, Joyce and Irving Goldman Medical School, Ben-Gurion University of the Negev, Beer Sheva, Israel

**Keywords:** HFrEF, HFimpEF, improved EF, prognosis, GDMT

## Abstract

**Background:**

Heart failure with improved ejection fraction (HFimpEF) is a recently recognized entity presenting a diagnostic and therapeutic challenge. Our aim was to characterize the profile of HFimpEF patients and evaluate predictors for EF lack of improvement among heart failure with reduced ejection fraction (HFrEF) patients.

**Methods:**

We included ambulatory HFrEF patients (EF≤40%) between January 1, 2015, and September 1, 2022, with two consecutive echocardiography exams at least 6 months apart. HFimpEF was defined as improved EF from ≤40%–>40% and by ≥10%.

**Results:**

A total of 567 HFrEF patients (72% male, 54.3 ± 14.4 years old) were analyzed. Patients without EF improvement were more likely to be male, had more comorbidities, ischemic cardiomyopathy (ICMP), markers of adverse cardiac remodeling (lower EF and higher left and right ventricular diameters) and presence of late gadolinium enhancement (LGE) in MRI (*P* < 0.05 for all). In a multivariate analysis, male sex, ICMP, lower EF, larger ventricular size and LGE remained independent predictors for lack of EF improvement. A prediction model for lack of EF improvement including LVEF, LV diameter, diastolic blood pressure and ischemic etiology exhibited an area under the ROC curve of 0.77 (95% CI 0.73–0.81; *P* < 0.001). HFimpEF patients had better prognosis with lower hospitalizations and mortality rates. Guideline directed medical therapy (GDMT) were associated with improved outcomes in both groups regardless of EF improvement.

**Conclusions:**

Lack of improvement in EF among HFrEF patients may be predicted by HF etiology and imaging parameters of adverse cardiac remodeling, and is associated with worse prognosis. GDMT were associated with improved outcomes in both HFimpEF and HFrEF patients.

## Introduction

Although our comprehension of the fundamental mechanisms underlying heart failure (HF) disease has improved, patients with HF are still traditionally classified based on their left ventricular ejection fraction (LVEF) due to differing prognosis and response to treatment (1, 2). The progress made in pharmacological and device therapy for HF has led to an increase in patients with a significant improvement in LVEF (2). Despite being associated with better outcomes compared to HF with reduced EF (HFrEF) patients (3), an improved LVEF does not necessarily indicate full myocardial recovery or normalization of left ventricular (LV) function. Therefore, the American Heart Association 2022 guidelines for the management of HF have introduced “HF with improved EF” (HFimpEF) as a subgroup of HFrEF to better characterize these patients (1).

Previous studies focused on clinical determinants of HFimpEF have identified several potential predictors for lack of EF improvement, including older age, male sex, more comorbidities, ischemic etiology, longer duration of HF and severe adverse cardiac remodeling (4–7). Discerning patients who are more likely to improve their EF from those who won't, is of great importance and may guide therapeutic decisions, as despite having generally better clinical outcomes and lower mortality, HFimpEF patients still experience HF symptoms and adverse events (3). Moreover, there is little or no consensus regarding the definition, diagnosis, and management of this growing HF population. The contemporary European Society of Cardiology Guidelines do not provide sufficient information on how to define and manage patients with an improvement in EF (2), while the American Heart Association Guidelines only suggest maintenance of guidelines-directed medical therapy(GDMT) recommended for HFrEF (1).

Therefore, the objective of this study was to characterize the profile, clinical course and response to medical therapy of HFimpEF patients compared with HFrEF patients in a contemporary real-life cohort, as well as to identify predictors for EF lack of improvement within a group of chronic ambulatory HFrEF patients.

## Methods

We conducted a retrospective analysis of all ambulatory HFrEF patients, identified by a diagnosis of HF or related ICD-10 code and LVEF ≤40%. All patients were followed at the outpatient cardiovascular clinics of the Sheba Medical Center, between January 1, 2015, and September 1, 2022. The study cohort included only patients with two consecutive echocardiography exams, separated by a minimum interval of 6 months, performed and analyzed at the Sheba medical center echocardiography lab and were interpreted by multiple echocardiography specialists. The most commonly used apparatuses were the Philips Epiq 7 and Philips Affinity 70. LVEF improvement was defined as an increase from a baseline EF value of ≤40%–>40% and by an absolute increase of ≥10% (1, 4). Patients were stratified into two groups based on EF improvement.

The Institutional Review Board of the Sheba Medical Center approved this study on the basis of strict maintenance of participants’ anonymity during database analyses. No individual consent was obtained. Patient and Public were not involved in the research design or performance. The data underlying this article will be shared on reasonable request to the corresponding author.

### Clinical data

Clinical and echocardiographic data were automatically extracted from the electronic medical record. Laboratory data were obtained from the closest available exam within 3 months of the corresponding echocardiography exam. Right ventricle (RV) enlargement was defined as a basal diameter of >35 mm, according to the ASE guidelines (8). RV systolic function was determined by visual estimation. Cardiac magnetic resonance (CMR)was performed in various laboratories across the country, and the data was manually extracted from the closest available exam within 3 months of the first echocardiography. Medications were included if prescribed within 3 months of the corresponding echocardiography. Outcome events used in this study included hospitalizations at the Sheba medical center and all-cause mortality. Mortality data was extracted from the Israeli National Population Registry and was available for all cases.

### Validation and quality assurance

The medical records of all patients were manually examined. The etiology of HF was obtained from the medical record based on the physician's clinical judgment. CMR data were all manually retrieved. All records were scrutinized for accuracy and completeness of the data.

### Statistical analysis

Descriptive statistics were employed to analyze the attributes of the study population. Each variable was presented by the most suitable central and dispersion measures: dichotomous and nominal variables were presented by mode (%), numerical (continuous) variables by mean ± standard deviation (SD) and numerical (count) variables by median and inter-quartile range (IQR). For each variable we assessed the number of missing values. No Imputation of missing data was conducted.

First, we conducted univariate analysis to examine the baseline characteristics in both study groups (improved vs. non-improved EF). To analyze nominal variables, we utilized either the chi-square test or the Fisher exact test. For continuous variables with a normal distribution, we used the *T*-test. For count variables and continuous variables with a non-normal distribution, we employed the Mann-Whitney test. Subsequently, we evaluated the association between patients’ clinical status, medication use, and echocardiographic results with EF improvement through logistic regression analysis. For each variable we carried out both univariable and multivariable regression analysis that was adjusted for potential clinical and sociodemographic confounders that had demonstrated a significant association in our univariate analysis. In addition, we created a prediction model for the clinical factors associated with non-improved EF, using the receiver operating characteristics (ROC) analysis and Youden index method to define the optimal cutoff value. The model is based on the clinical variables which showed significant association with patient lack of improvement in the multivariable regression analysis. We assessed collinearity between independent variables, excluding variables with strong collinearity. Relevant variables were entered into the model using the stepwise method and model quality was assessed using appropriate tests and AUC. Also, we investigated the association between medication use and the readmission and mortality rate by utilizing Poisson regression and logistic regression, respectively. Eventually, we used Kaplan-Meier curves with log-rank test as well as univariable Cox proportional-hazard regression to assess the hazard ratio (HR) of patients with improved vs. non-improved EF as well as among patients taking over three GDMT, without adjusting for other variables.

All analyses were conducted using IBM SPSS Statistics for Windows, Version 25.0. Armonk, NY, USA: IBM Corp and R Foundation for Statistical Computing, Vienna, Austria. A two-sided test significance level of 0.05 was used throughout the entire study.

## Results

### Baseline patients’ characteristics

The study cohort included 567 HFrEF patients (72% male, 54.3 ± 14.4 years old) with a mean baseline EF of 26.9 ± 8.8%; 174 patients (31%) improved their EF to >40% and by an absolute increase of ≥10% (Supplementary Figure S1). Patients in the non-improved EF group were more likely to be male, had more comorbidities including hypertension (HTN), chronic kidney disease (CKD) and dyslipidemia and had lower systolic and diastolic blood pressure compared with the improved EF group (Table 1). In addition, patients in the non-improved EF group had higher rates of ischemic cardiomyopathy (ICMP), while those in the improved EF group manifested higher incidence of dilated cardiomyopathy (DCMP) and arrhythmia induced cardiomyopathy (AICMP; *p* < 0.001 for all). There were no statistically significant differences observed between the two study groups in terms of age or New York Heart Association (NYHA) functional class. At baseline, a statistically significant disparity in medical therapy was observed between the two groups. The non-improved EF group had a higher usage of beta blockers, angiotensin receptor-neprilysin inhibitors (ARNI), diuretics, statins, and digoxin. Baseline echocardiographic parameters also differed significantly between the groups. The non-improved EF group demonstrated a lower LVEF, while the left ventricular end-diastolic dimension (LVEDD), left ventricular end-systolic dimension (LVESD), left atrial volume index (LAVi), and systolic pulmonary artery pressure (SPAP) were higher as compared to the improved EF group. Furthermore, the RV was more frequently observed to be dilated in the non-improved EF group. CMR data were available for 18% of patients (*n* = 100; 46 in the improved-EF group and 56 in the not-improved EF group) and revealed similar findings, where the non-improved EF group had lower LVEF and RVEF and higher LV volumes compared to HFimpEF patients. Moreover, the non-improved EF group exhibited a higher prevalence of late gadolinium enhancement (LGE) in a qualitative assessment. The percentage of LGE was missing for a majority of exams. However, for a subset of patients (12% of the total cohort) data regarding the LGE pattern was available. The presence of an ischemic LGE pattern was not statistically significantly different between the groups.

**Table 1:** Baseline patients’ characteristics.

	Total (*n* = 567)	Improved (*n* = 174)	Not improved (*n* = 393)	*P*
Age—yr.	54.3 ± 14.4	55.1 ± 14.0	53.9 ± 14.6	0.394
Male sex	409 (72.1)	104 (59.8)	305 (77.6)	**<0** **.** **001**
BMI—kg/m^2^	27.9 ± 5.5	28.2 ± 6.2	27.8 ± 5.1	0.409
Atrial fibrillation	187 (33.0)	59 (33.9)	128 (32.6)	0.755
Diabetes	239 (42.2)	64 (36.8)	175 (44.5)	0.085
Dyslipidemia	416 (73.4)	112 (64.4)	304 (77.4)	**0**.**001**
Hypertension	300 (54.2)	83 (47.7)	217 (57.1)	0.039
CKD	105 (18.5)	23 (13.2)	82 (20.9)	**0**.**031**
HF duration—months.	6.7 ± 19.4	4.9 ± 16.6	7.5 ± 20.5	**0**.**018**
Heart failure etiology				
Ischemic CMP	262 (46.2)	37 (21.3)	225 (57.3)	**<0**.**001**
DCMP	180 (31.7)	82 (47.1)	98 (24.9)	**<0**.**001**
Arrhythmia induced CMP	18 (3.2)	13 (7.5)	5 (1.3)	**<0**.**001**
Post chemotherapy	26 (5.4)	11 (6.3)	15 (4.9)	0.503
Valvular CMP	44 (7.8)	12 (6.9)	32 (8.1)	0.609
Infiltrative disease	14 (2.5)	5 (2.9)	9 (2.3)	0.680
NYHA class >2	196 (41.1)	48 (37)	148 (43)	0.229
Systolic BP	121.2 ± 21.9	126.0 ± 23.5	119.1 ± 20.8	**0**.**003**
Diastolic BP	71.8 ± 14.3	75.0 ± 16.4	70.4 ± 13.0	**0**.**010**
Heart rate	78.0 ± 17.8	79.8 ± 16.7	77.1 ± 16.9	0.161
PR interval—msec	167.7 ± 38.2	163.3 ± 37.4	169.4 ± 38.4	0.064
QRS duration—msec	119.0 ± 33.7	114.8 ± 32.1	120.8 ± 34.2	0.052
QRS>120 msec	234 (41.3)	60 (34.5)	174 (44.3)	**0**.**029**
Correct Qt interval—msec	467.9 ± 40.5	468.8 ± 54.8	468.6 ± 50.8	0.847
Hemoglobin^a^—g/dl	12.8 ± 2.2	12.7 ± 2.0	12.8 ± 2.3	0.446
Creatinine^a^—mg/dl	1.2 ± 0.7	1.1 ± 0.7	1.2 ± 0.7	0.06
Albumin^a^—g/dl	3.7 ± 0.5	3.7 ± 0.5	3.7 ± 0.5	0.353
LVEF—%	26.9 ± 8.8	29.7 ± 8.4	25.6 ± 8.7	**<0**.**001**
LVEDD—mm	5.8 ± 0.8	5.5 ± 0.7	5.9 ± 0.9	**<0**.**001**
LVESD—mm	4.9 ± 2.9	4.4 ± 0.8	5.1 ± 3.4	**<0**.**001**
LAVi- ml/m^2^	44.0 ± 35.3	36.7 ± 12.2	46.7 ± 40.4	**0**.**002**
MR≥moderate	211 (40.8)	61 (39.9)	150 (41.2)	0.777
TR≥moderate	117 (22.7)	30 (19.6)	87 (24)	0.273
SPAP^b^—mmHg	42.2 ± 14.3	39.2 ± 13.2	43.4 ± 14.6	**0**.**007**
Enlarged right ventricle	109 (33.9)	22 (23.6)	87 (38.2)	**0**.**013**
RV systolic dysfunction	186 (55.8)	46 (49)	140 (58.5)	0.11
CMR^c^				
LVEF-%	32.2 ± 11.2	35.5 ± 12.4	29.5 ± 9.3	**0**.**010**
LVEDVi	112.4 ± 35.1	103.4 ± 33.5	119.6 ± 35.0	**0**.**005**
LVESVi	78.3 ± 34.2	68.3 ± 31.2	86.4 ± 34.5	**0**.**002**
RVEF	44.2 ± 14.2	48.7 ± 14.6	41.0 ± 13.2	**0**.**015**
RVEDVi	87.6 ± 40.4	82.9 ± 31.6	91.1 ± 45.9	0.595
RVESVi	51.8 ± 33.3	45.9 ± 27.9	56.1 ± 37.4	0.171
LGE	70 (70)	25 (54.3)	45 (83.3)	**0**.**002**
Ischemic LGE pattern^d^	17 (25)	5 (20)	12 (27)	0.5
Beta blockers	525 (92.6)	147 (84.5)	378 (96.2)	**<0**.**001**
ACE inhibitors	449 (79.2)	134 (77.0)	315 (80.2)	0.395
Aldactone	340 (60)	92 (52.9)	248 (63.1)	**0**.**022**
Furosemide	384 (67.7)	95 (54.6)	289 (73.5)	**<0**.**001**
Statin	352 (62.1)	85 (48.9)	267 (67.9)	**<0**.**001**
Amiodarone	105 (18.5)	27 (15.5)	78 (19.8)	0.221
Digoxin	62 (10.9)	11 (6.3)	51 (13.0)	**0**.**019**
ARNi	72(12.7)	9(5.2)	63(16.0)	**<0**.**001**
SGLT2i	65(11.5)	14(8.0)	51(13.0)	0.089

Data are expressed as mean ± SD, or median (interquartile range) or numbers (%), when appropriate.

BMI, body mass index; BP, blood pressure; CKD, chronic kidney disease; DCMP, dilated cardiomyopathy; LGE, late gadolinium enhancement; LVEF, left ventricular ejection fraction; LVEDD/LVESD, left ventricular end-diastolic/systolic diameter; MR, mitral regurgitation; NYHA, New York heart association; SPAP, systolic pulmonary artery pressure; TR, tricuspid regurgitation.

^a^ Missing for 135 (24%) of patients.

^b^ Missing for 130 (23%) of patients.

^c^ Missing for 467 (82%) of patients.

^d^ Missing for 499 (78%) of patients.

Bold values denote statistically significant values.

### Patient characters at follow-up echocardiography

At the time of the second echocardiographic evaluation (1.3 ± 1.2 years from the first echocardiography exam, maximum 7.3 years; Supplementary Table S1), the LVEF was 50 ± 6% in the improved EF group vs. 26 ± 8% in the non-improved EF group (LVEF change from baseline echocardiography by 20.3 ± 10.8% vs. 0.84 ± 7.6%, respectively; *P* < 0.001). The non-improved EF group had worse NYHA functional class compared to HFimpEF group (*p* < 0.001). Medication use showed a similar trend with higher usage rates of angiotensin-converting enzyme inhibitors (ACE) or angiotensin receptor blockers (ARB), ARNI, diuretics, mineralocorticoid receptor antagonists (MRA), statins and digoxin in the non-improved EF group. Moreover, the non-improved EF group exhibited higher LVEDD, LVESD, LAVi and SPAP measurements, alongside increased rates of mitral regurgitation (MR) and tricuspid regurgitation (TR), dilation of RV and reduced RV function (*p* ≤ 0.001 for all). There were no significant differences between the groups in the rate of implantable cardioverter-defibrillator (ICD) or cardiac resynchronization therapy (CRT) implantation between the first and follow-up echocardiography. Additionally, there were no significant differences between the groups in the rate of revascularization therapy (percutaneous or surgical), valvular interventions or electrophysiologic interventions between the first and follow-up echocardiography (Supplementary Table S1).

### Parameters associated with lack of EF improvement

**Figure 1:**
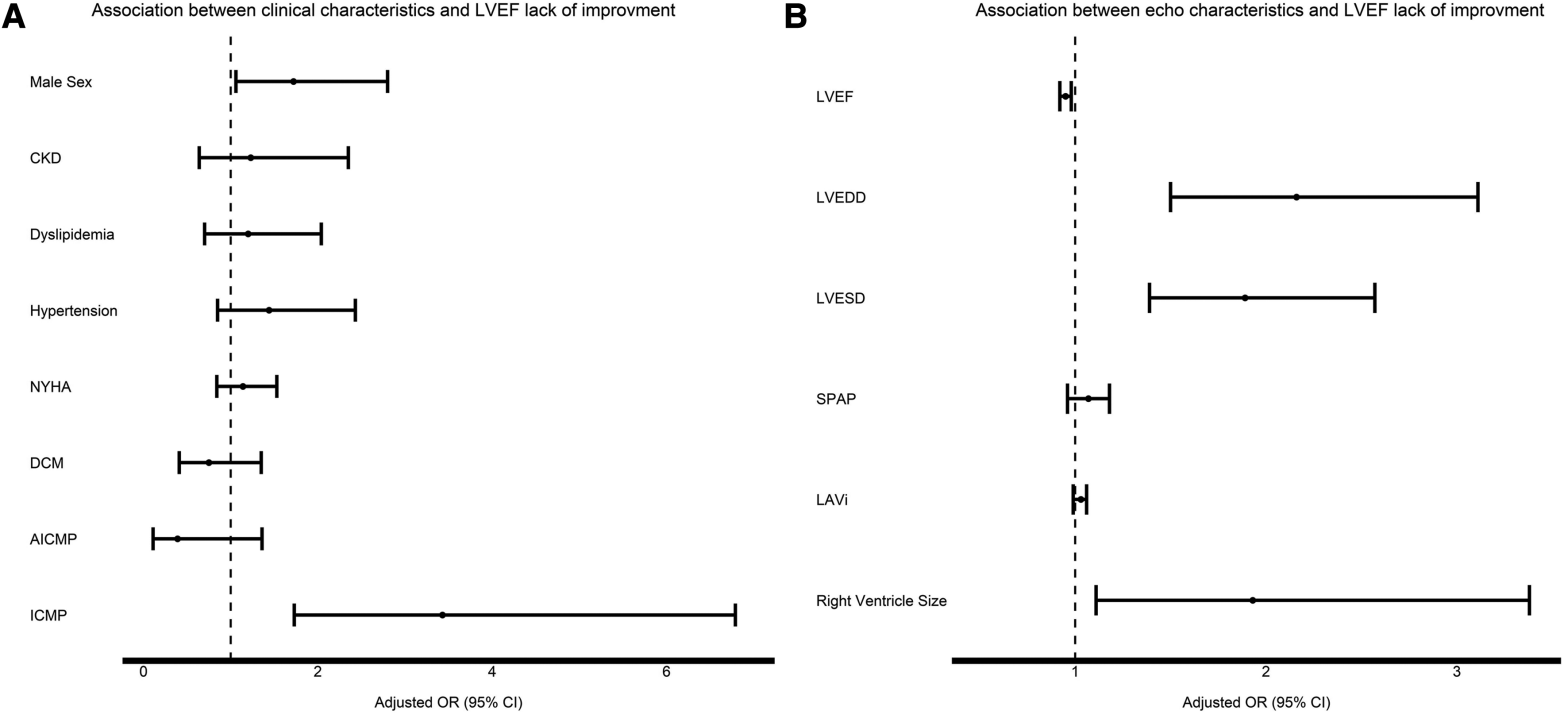
Association between clinical characteristics (**A**) and echocardiographic characteristics (**B**) to EF improvement. For each variable the black square signifies the adjusted OR and the whiskers signify the 95% confidence interval. OR > 1 means increased risk for EF lack of improvement.

Table 2 illustrates the clinical and imaging (echocardiographic and CMR) features associated with lack of EF improvement. Male sex, the presence of comorbidities (HTN, CKD, dyslipidemia), systolic and diastolic blood pressure, HF etiology, NYHA functional class at baseline, medication use, LV function and size (at both echocardiography and CMR), LAVi, SPAP, RV size, the presence of LGE and RVEF in CMR were all determinates of lack of EF improvement in univariate analysis. In a multivariable analysis, adjusted for all clinical parameters that had demonstrated a significant association in the univariate analysis, male sex, ICMP, the use of beta blockers, diuretics, statins and ARNI, as well as LV function and size, RV size and the presence of LGE and RVEF in CMR were found to be independently associated with lack of EF improvement (Table 2 and Figure 1).

**Table 2:** Variables associated with LVEF lack of improvement.

	Univariate analysis			Multivariate analysis^a^		
	OR	95% CI	*P* value	Adjust. OR	95% CI	*P* value
Male sex	2.33	1.59–3.43	**<0**.**001**	1.72	1.06–2.80	**0**.**028**
Dyslipidemia	1.89	1.28–2.79	**0**.**001**	1.20	0.70–2.04	0.516
Hypertension	1.46	1.02–2.09	**0**.**04**	1.44	0.85–2.43	0.179
CKD	1.73	1.05–2.86	**0**.**032**	1.23	0.64–2.35	0.536
HF duration—months	1.01	0.99–1.02	0.146	1.01	0.99–1.02	0.157
ICMP	4.96	3.28–7.51	**<0**.**001**	**3**.**43**	**1.73–6.79**	**<0**.**001**
AICMP	0.16	0.06–0.46	**0**.**001**	0.39	0.11–1.36	0.139
DCM	0.37	0.26–0.54	**<0**.**001**	0.75	0.41–1.35	0.332
NYHA	1.35	1.04–1.76	**0**.**026**	1.14	0.84–1.53	0.398
Systolic BP	0.99	0.98–0.99	**0**.**001**	0.98	0.97–0.99	**0**.**026**
Diastolic BP	0.97	0.97–0.99	**0**.**001**	0.99	0.97–1.02	0.850
LVEF	0.94	0.92–0.97	**<0**.**001**	0.95	0.92–0.98	**0**.**001**
LVEDD	1.86	1.47–2.36	**<0**.**001**	2.16	1.50–3.11	**<0**.**001**
LVESD	1.62	1.34–1.97	**<0**.**001**	1.89	1.39–2.57	**<0**.**001**
LAVi	1.03	1.01–1.06	**0**.**001**	1.03	0.99–1.06	0.066
SPAP	1.02	1.01–1.04	**0**.**007**	1.07	0.98–1.18	0.135
Right ventricle size	1.56	1.12–2.19	**0**.**009**	1.93	1.11–3.38	**0**.**02**
MRI LVEF	0.95	0.91–0.99	**0**.**011**	0.95	0.89–1.01	0.081
MRI LVEDVi	1.02	1.01–1.03	**0**.**031**	1.03	1.01–1.05	**0**.**035**
MRI LVESVi	1.02	1.01–1.04	**0**.**014**	1.03	1.01–1.05	**0**.**037**
MRI RVEF	0.96	0.93–0.99	**0**.**019**	0.92	0.86–0.98	**0**.**014**
MRI LGE	4.20	1.67–10.55	**0**.**002**	10.39	1.95–55.25	**0**.**006**

AICMP, arrhythmia induced cardiomyopathy; BP, blood pressure; CKD, chronic kidney disease; DCM, dilated cardiomyopathy; ICMP, ischemic cardiomyopathy; LAVi, left atrial volume index; LVEF, left ventricular ejection fraction; LVEDD/LVESD, left ventricular end-diastolic/systolic diameter; LVEDVi/LVESVi, left ventricular end diastolic/systolic volume index; SPAP, systolic pulmonary artery pressure.

^a^ Multivariable Logistic Regression, adjusted for patient sex, patients’ significant comorbidities (hypertension, CKD, dyslipidemia), HF etiology (DCMP, arrythmia induced CMP, ischemia), NYHA Class at 1st echo, systolic BP at 1st echo, and time from diagnosis to 1st echo.

Bold values denote statistically significant values.

### Prediction model

Based on our results as described above, we formed a prediction model for lack of EF improvement among HFrEF patients. The model includes baseline LVEF, LV end-diastolic diameter, and systolic BP, all as continuous variables, and the presence of an ischemic etiology (Supplementary Table S2). The model exhibited an area under the ROC curve of 0.77 (95% CI 0.72–0.81; *P* < 0.001), a sensitivity of 73% and specificity of 69% (Supplementary Figure S2).

### Clinical outcomes

**Figure 2:**
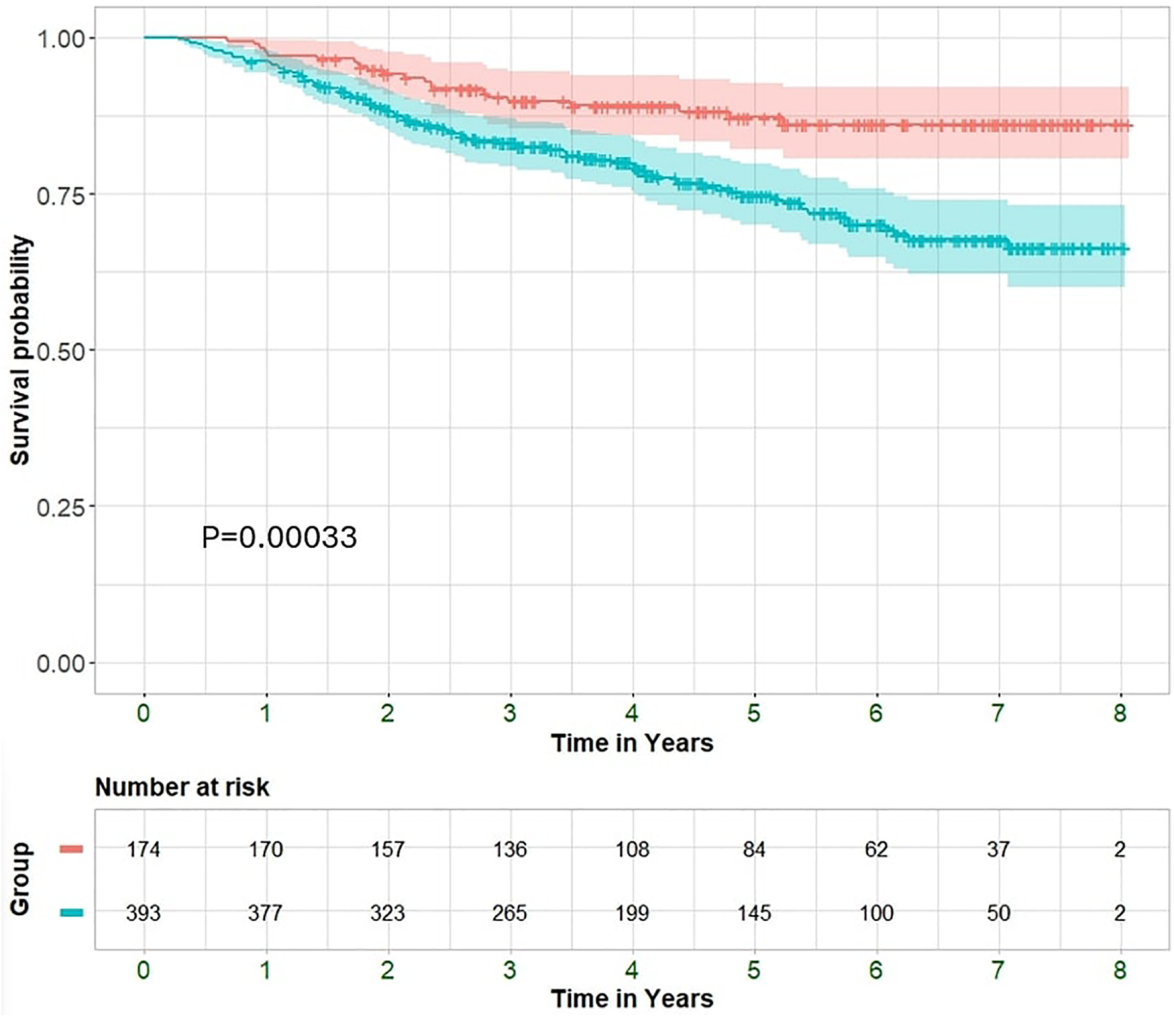
Kaplan–Meier curves displaying cumulative survival probability with 95% confidence interval among improved and non-improved patients.

Over a mean follow up of 4.4 ± 2.1 years, a total of 296 (53%) were hospitalized and 118 (21%) patients died. The non-improved EF group exhibited increased rates of hospitalizations compared with the HFimpEF group (246 (63%) vs. 50 (31%); *P* < 0.001 and median 1, IQR 1-2 vs. 0, IQR 0-1; *P* < 0.001) and all-cause mortality (24% vs. 13%; *P* = 0.001) (Figure 2). Moreover, the non-improved EF group had worse functional class at the time of the second echocardiography exam (NYHA>2 at 41% vs. 16%; *P* < 0.001).

**Figure 3:**
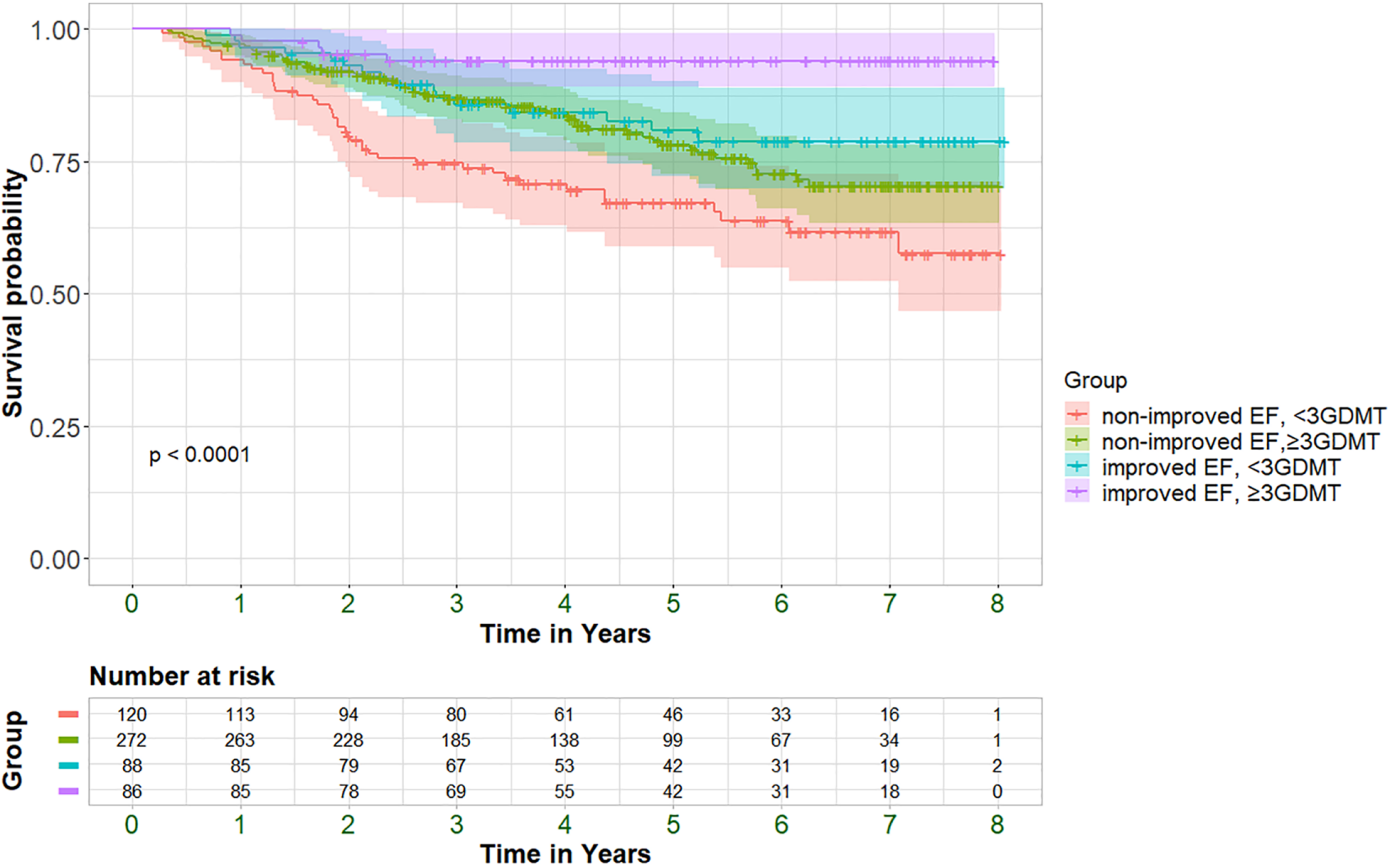
Kaplan–Meier curves displaying cumulative survival probability with 95% confidence interval (95%CI) among improved+/-≥3GDMT and non-improved +/-≥3GDMT patients. *P* = pv of log-rank test.

The association between medication usage and hospitalization and mortality rates was assessed. All GDMT displayed a trend towards decreased mortality in the entire cohort, while statistically significant reductions in mortality rates were observed with the usage of MRA, SGLT2i, ARNI and the use of a combination of at least three GDMT. Similar trends were also observed for hospitalization rates (Supplementary Figure S3). While the effect of reduced mortality rates among patients taking ≥3 GDMT was consistent irrespective of EF improvement status, the magnitude of the decrease was larger in the improved-EF group compared to the non-improved group (HR 0.32, 95% CI 0.12–0.86; *P* = 0.024 vs. HR 0.58, 95% CI 0.39–0.88; *P* = 0.009, respectively). The survival of the entire cohort, stratified by EF improvement status as well as GDMT status, is depicted in Figure 3. Our analysis revealed a graded effect, with the worse survival observed in patients without EF improvement who did not receive ≥3 GDMT, followed by those without EF improvement but taking ≥3 GDMT. HFimpEF patients not taking ≥3 GDMT had better survival, while those with improved EF and ≥3 GDMT had the best survival.

## Discussion

The present study outlines the clinical characteristics associated with EF improvement and offers a prediction model for such improvement in a sizable real-life HFrEF cohort of chronic outpatients. Moreover, we report worse prognosis with higher rates of all-cause mortality and hospitalizations within HFrEF patients who did not improve their EF, as compared to those who did. Interestingly, the use of GDMT was not associated with EF improvement but did manifest a significant decrease in mortality and hospitalization rates in both groups regardless of EF.

To the best of our knowledge, this is the most comprehensive study thus far to include a wide spectrum of variables within the same cohort, as well as propose a prediction model for the occurrence of HFimpEF. Moreover, this study focuses on chronic ambulatory HFrEF patients, where the initial reduction in EF cannot be attributed to an acute or transient process. Furthermore, a strict definition of improved EF, that is in line with current consensus (4), was employed in this contemporary cohort that included patients treated with the most updated GDMT including ARNI and SGLT2i.

### Predictors for EF improvement

Similarly to previous publications, we have found in the non-improved EF group higher proportion of male and ICMP patients, along with longer HF duration, more comorbidities and imaging markers of adverse cardiac remodeling (4, 5, 7). Female sex has previously been associated with an increased likelihood of EF improvement, possibly due to better response to CRT implantation (9) and lower rates of ICMP. However, in our cohort, there were no significant differences in CRT implantation rates between the groups, and the effect of sex on EF improvement remained significant even after a multivariate analysis correcting for the HF etiology. These findings highlight the gap in our knowledge and understanding of the mechanism behind sex related differences in HF physiology. In terms of etiology, ICMP manifested lower probability of EF improvement, compared to DCM and AICMP. These differences may be explained by the irreversible myocardial ischemic scar, which reduces the likelihood of EF improvement over time, but may provide a possible explanation for the well-established worse prognosis associated with ICMP compared to non-ischemic CMP (10).

In previous studies, patients with improved EF had better NYHA functional class (3). However, there was no significant difference in baseline NYHA class between both groups in our cohort, nor NYHA was associated with EF improvement in a multivariate model. This finding is clinically important because it suggests that functional class may only be a marker of HF severity.

Our analysis of cardiac imaging data yielded similar results for both echocardiography and CMR exams. We found that lower LVEF and higher LV size at baseline were independent predictors for lack of EF improvement in a multivariate analysis, consistent with previous studies (4). Furthermore, the presence of LGE in a qualitative assessment was identified as a strong predictor of lack of EF improvement, similar to previous reports on both ischemic and nonischemic CMP (11–13). The presence of an ischemic LGE pattern was numerically higher in the non-improved group. This finding is consistent with the higher rate of ischemic etiology in this group; however, it did not reach statistical significance, likely due to the small sample size. Our data reinforces the significance of adverse cardiac remodeling as strong and independent predictors associated with lack of EF improvement. Compared to previous studies, a significant strength of our study is the comprehensive assessment of various parameters, encompassing detailed clinical, echocardiographic, and medication data, within a single cohort. CMR data was available for a substantial minority of the cohort and adds another layer to the existing imaging data.

To better inform patients and physicians and to further refine the echocardiographic and clinical prognosis of patients with HFrEF, we also constructed a prediction model for EF lack of improvement, using LVEF, LVEDD, systolic blood pressure and ischemic etiology. While this model is comprised of simple readily-available parameters, it exhibited superior performance compared to recent more complicated ones (14), and may be of significant clinical utility.

### GDMT and EF improvement

Previous randomized studies have demonstrated the reverse cardiac remodeling effect of GDMT. The use of beta blockers, ACE inhibitors, ARB's and MRA's were each associated with an EF improvement by approximately 5% (15). In the PROVE-HF prospective observational single-group, open-label study of patients with HFrEF, the use of ARNI for 12 months was associated with a statistically significant 9.4% increase in mean LVEF (LVEF increased from 28.2% to 37.8%) (15). Importantly, the magnitude of EF improvement in the aforementioned trials does not qualify for the updated criteria for HFimpEF in recent guidelines as applied in the current study (an increase from a baseline EF value of ≤40% to >40% and by an absolute increase of ≥10%) (1, 4). In addition, while no significant differences were observed in the rates of above MR or TR, nor in the SPAP value between the groups at baseline, follow-up echocardiography revealed a lower rate of these valvular abnormalities and a reduction in SPAP in the improved group. This finding aligns with the reverse remodeling process observed in patients with improved EF. Interestingly, the group without EF improvement, despite receiving high rates of GDMT, maintained similar rates of valvular abnormalities, and their SPAP remained unchanged, indicating a lack of reverse remodeling in this group. In our cohort, there was no association between the use of GDMT and EF improvement. On the contrary, we found an inverse relation, such that patients without EF improvement were treated more often with GDMT, as compared to those with EF improvement. This finding may in part be explained by the worse cardiac remodeling parameters and higher rates of comorbidities in the non-improved EF group mandating more intensive medical treatment. Furthermore, the fact that both study groups received adequate medical therapy at baseline may have mitigated the potential effect of GDMT on EF improvement. Nevertheless, our findings are consistent with several previous non-randomized studies including a total of over 5,000 HFrEF patients, where no association was found between GDMT and EF improvement (3, 14, 16). These studies corroborate our results and emphasize the significant effect of clinical parameters and cardiac remodeling on the likelihood of EF improvement, rather than a specific medication.

Moreover, in our study GDMT was associated with improved clinical outcomes in the entire cohort, including lower hospitalization and mortality rates. This observation supports the well-established role of GDMT in reducing clinical adverse events irrespective of EF improvement and suggests that the mechanism underlying this reduction may not be dependent on EF improvement. Furthermore, we observed an even greater survival benefit among patients with improved EF who received GDMT, which provides additional support for the less established role of GDMT among HFimpEF patients.

Interestingly, there was no difference in the CRT implantation rate between the groups. This may be explained by the fact that only a minority of patients improve their EF to over 50% following CRT implantation (17), which was the mean EF in the improved group in our study. The observed differences in the rate of wide QRS among the groups suggest that wide QRS may be a marker of disease severity, reflecting the worse functional and structural abnormalities identified in cardiac imaging studies among patients without EF improvement.

Patients in the non-improved EF group had worse functional status, higher rates of hospitalizations and most importantly, higher all-cause mortality rates. These findings are consistent with previous reports (3, 4, 18) and highlight the worse prognosis of HFrEF patients who do not improve their EF, compared to those who do.

## Limitations

We acknowledge several limitations to our study. First, this is an observational study with a retrospective analysis of collected data. Second, the study's generalizability may be limited as it was conducted in the outpatient clinics of a single tertiary medical center, which could result in patient selection bias. However, our intentional focus on ambulatory patients enabled us to evaluate the potential for improvement in a stable setting, remote from the acute event, and thus enhancing the clinical impact of our findings. In addition, since EF is visually estimated, changes between exams may be attributed to interobserver variability rather than a true change in EF. Nonetheless, all exams were conducted at the same echocardiography lab, and we set a criterion of an absolute increase >10%, thereby reducing this potential bias, and visually estimated LVEF is the actual measurement used to guide patient care in many centers. Furthermore, CMR data was available for only a limited number of patients and should therefore be considered supplementary. However, for those patients with available data, MRI findings were consistent with the echocardiography and clinical findings in terms of chamber function, size, and etiology as evidenced by the LGE pattern. Moreover, despite the high rate of GDMT use in our cohort, the utilization of SGLT2i and ARNI was comparatively low. Nonetheless, this rate was significantly greater than prior contemporary studies (14). Lastly, rehospitalizations were considered in our center alone. However, the hospitalization rate in our cohort was significant (53% in the entire cohort were hospitalized during a mean follow-up period of 4.4 ± 2.1 years; 246 (63%) in the not-improved vs. 50 (31%) in the improved group; *P* < 0.001). This rate is not significantly different from the expected hospitalization rate in the corresponding HF population [22% and 32% in one year for HFmrEF/HFpEF and HFrEF patients respectively (19), and −80% in 5 years for both (20)].

## Conclusions

Among a contemporary cohort of ambulatory HFrEF patients, improvement in EF may be predicted by baseline clinical characteristics and imaging parameters of adverse cardiac remodeling. A prediction model comprised of LVEF, LVEDD, systolic blood pressure and ischemic etiology may identify patients who are less likely to improve. Although EF improvement is associated with better outcomes, GDMT improve clinical outcomes in patients with and without EF improvement.

Our findings contribute to the growing published data on HFimpEF and further validates the role of GDMT in reducing clinical outcomes, regardless of EF improvement.

## Data Availability

The raw data supporting the conclusions of this article will be made available by the authors, without undue reservation.

## Supplementary material

Supplementary Figure S1 Patients' flow chart.

Supplementary Figure S2 ROC curve for predicting no improvement in EF.

Supplementary Figure S3 Association between drug type to death (A) and number of hospitalizations (B). For each variable the square signifies the adjusted OR and the whiskers signify the 95% confidence interval. The OR for each drug is shown for the overall population (bright gray), the unimproved patients (gray) and the improved patients (black). OR>1 means increased factor for death/hospitalization.
