# Supplementary material for: Heart failure with improved ejection fraction: patient characteristics, clinical outcomes and predictors for improvement
Source: Front Cardiovasc Med. 2024 Jul 17;11:1378955. doi: 10.3389/fcvm.2024.1378955 (PMC11288926; doi:10.3389/fcvm.2024.1378955)
Supplement: Supplementary file 1 [file Table1.pdf]

Supplementary Table 1. Clinical character at follow-up echo				
	Total (n=567)	Improved (n=174)	Not Improved (n=393)	P
Age – yr.	61.6±14.2	61.6±14.7	63.5±14.1	0.155[a]
BMI - kg/m²	28.6±16.9	28.3±6.5	28.8±20.0	0.613
NYHA class >2	162(34.5)	19(15.6)	143(41.1)	**<0.001**
Heart Rate	72.2±13.9	70.9±15.6	72.8±13.1	**0.037**
Systolic BP	118.3±21.0	122.8±21.3	116.4±20.6	**0.002**
Diastolic BP	67.3±12.4	68.4±12.5	66.8±12.4	0.168
PR interval - msec	170.0±43.9	165.5±44.1	171.9±43.8	0.075
Hemoglobin	12.8±2.2	12.7±2.0	12.8±2.3	0.457
Creatinine	1.2±0.9	1.3±1.2	1.2±0.7	0.920
Albumin	3.7±0.5	3.8±0.5	3.7±0.5	0.232
LVEF - %	33.7±13.2	50.1±5.9	26.4±8.1	**<0.001**
LVEF Delta Change - %	6.8±12.5	20.3±10.8	0.84±7.6	**<0.001**
LVEDD – mm	5.6±0.8	5.2±0.6	5.9±0.8	**<0.001**
LVESD – mm	4.6±3.5	4.2±6.0	4.8±1.0	**<0.001**
LV mass index-g/m²	124.0±211.7	98.5±24.2	136.0±254.9	**<0.001**
LVIST – mm	1.0±0.5	1.0±0.2	1.0±0.6	0.139
LVPWT-mm	1.1±3.4	1.3±6.0	1.0±0.6	0.736
LAVi- ml/m²	40.2±18.8	35.3±16.8	42.8±19.3	**<0.001**
MR≥moderate	166(32.6)	26(17.5)	140(39)	**<0.001**

TR≥moderate	117(22.7)	18(11.8)	99(27.4)	**<0.001**
SPAP- mmHg	40.2±14.1	33.1±9.0	42.9±14.8	**<0.001**
Enlarged right ventricle	86(31.0)	11(15.7)	75(36.3)	**0.001**
RV systolic dysfunction	129(43.7)	4(5.7)	125(55.5)	**<0.001**
Beta blockers	518(91)	153(88)	365(93)	0.053
ACE inhibitors	399(70)	107(61)	292(74)	**0.002**
Aldactone	149(26)	34(19)	115(29)	**0.015**
Furosemide	403(71)	88(51)	315(80)	**<0.001**
Statin	367(65)	88(51)	279(71)	**<0.001**
SGLT2i	111(20)	27(15)	84(21)	0.105
Digoxin	62(11)	6(3)	56(14)	**<0.001**
Procedures done from 1st echo				
Any procedure	175(30.9)	59(33.9)	116(29.5)	0.296
CRT	72(12.7)	27(15.5)	45(11.5)	0.180
ICD	128(22.6)	41(23.6)	87(22.1)	0.708
PTCA	4(0.7)	2(1.1)	2(0.5)	0.590
CABG	6(1.1)	4(2.3)	2(0.5)	0.075
EP interventions	11(1.9)	6(3.4)	5(1.3)	0.083
TAVI	6(1.1)	3(1,7)	3(0.8)	0.377
MVR	5(0.9)	1(0.6)	4(1.0)	1.00
AVR	4(0.7)	2(1.1)	2(0.5)	0.590

Data are expressed as mean ± SD, or median (interquartile range) or numbers (%), when appropriate.

BP=blood pressure; CABG= coronary artery bypass grafting; CRT= cardiac resynchronization therapy; ICD=implantable cardioverter defibrillator; EP= electrophysiologic; LAVi= left atrium volume index; LVEDD/LVESD=left ventricular end-diastolic/systolic diameter; LVEF=left ventricular ejection fraction; LVIST=left ventricle interventricular septum thickness; LVPWT= left ventricle posterior wall thickness; MR=mitral regurgitation; MVR= mitral valve replacement; NYHA=New York heart association; PTCA= percutaneous transluminal coronary angioplasty; TAVI=transcatheter aortic valve implantation; TVR=tricuspid valve replacement; TR=tricuspid regurgitation; SPAP= systolic pulmonary artery pressure
