# Supplementary material for: Heart failure with improved ejection fraction: patient characteristics, clinical outcomes and predictors for improvement
Source: Front Cardiovasc Med. 2024 Jul 17;11:1378955. doi: 10.3389/fcvm.2024.1378955 (PMC11288926; doi:10.3389/fcvm.2024.1378955)
Supplement: Supplementary file 2 [file Table2.pdf]

**Supplementary Table 2 . Prediction model for clinical factors associated with non-Improved EF**			
	**Adjusted Odds Ratio**	**95%CI**	**p**
LVEF	0.96	0.94-0.99	**0.005**
LVEDD	1.61	1.20-2.15	**0.001**
Systolic BP	0.99	0.98-0.99	**0.005**
ICMP	5.27	3.39-8.20	**<0.001**

BP=blood pressure; ICMP=ischemic cardiomyopathy; LVEF=left ventricular ejection fraction;

LVEDD= left ventricular end-diastolic diameter
