# Supplementary figures and images for: Heart failure with improved ejection fraction: patient characteristics, clinical outcomes and predictors for improvement
Source: Front Cardiovasc Med. 2024 Jul 17;11:1378955. doi: 10.3389/fcvm.2024.1378955 (PMC11288926; doi:10.3389/fcvm.2024.1378955)

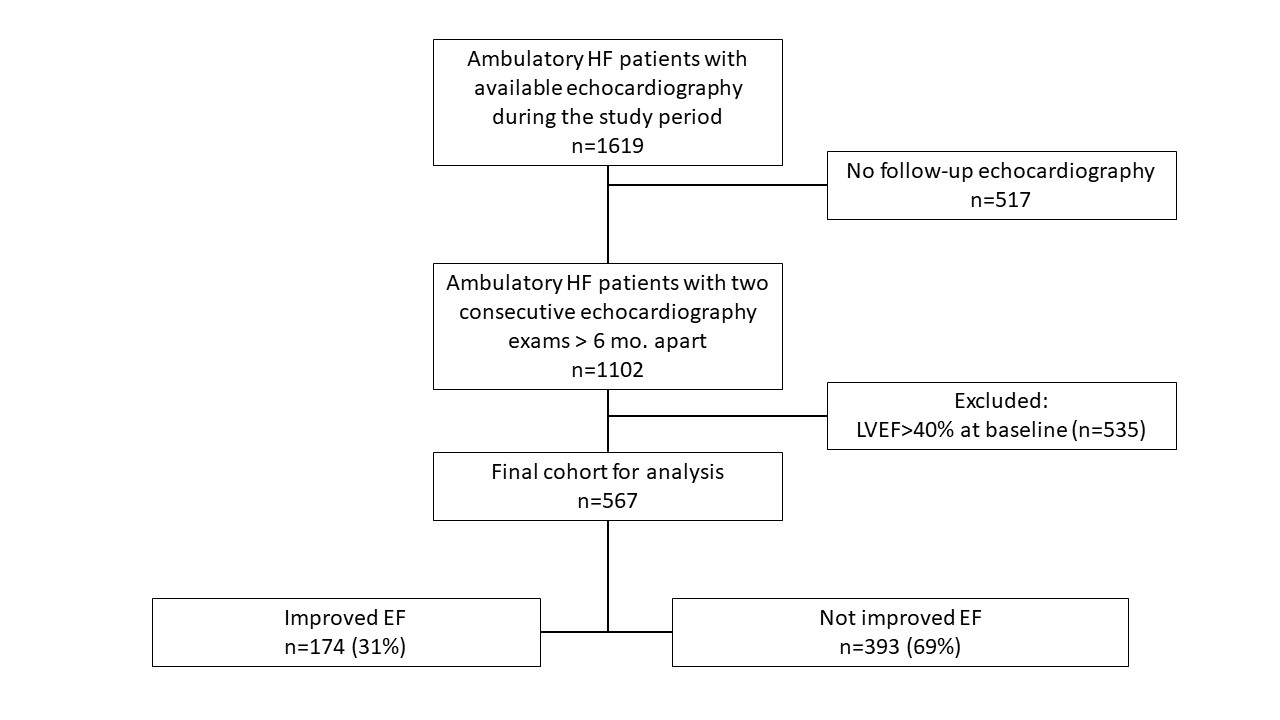

Supplement: Supplementary Figure S1 — Patients' flow chart. [file Image1.jpeg]

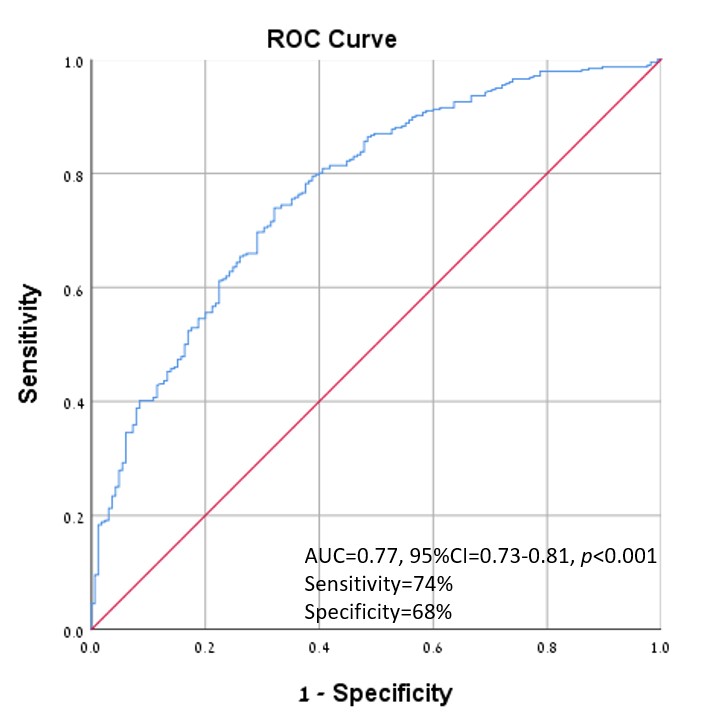

Supplement: Supplementary Figure S2 — ROC curve for predicting no improvement in EF. [file Image2.jpg]

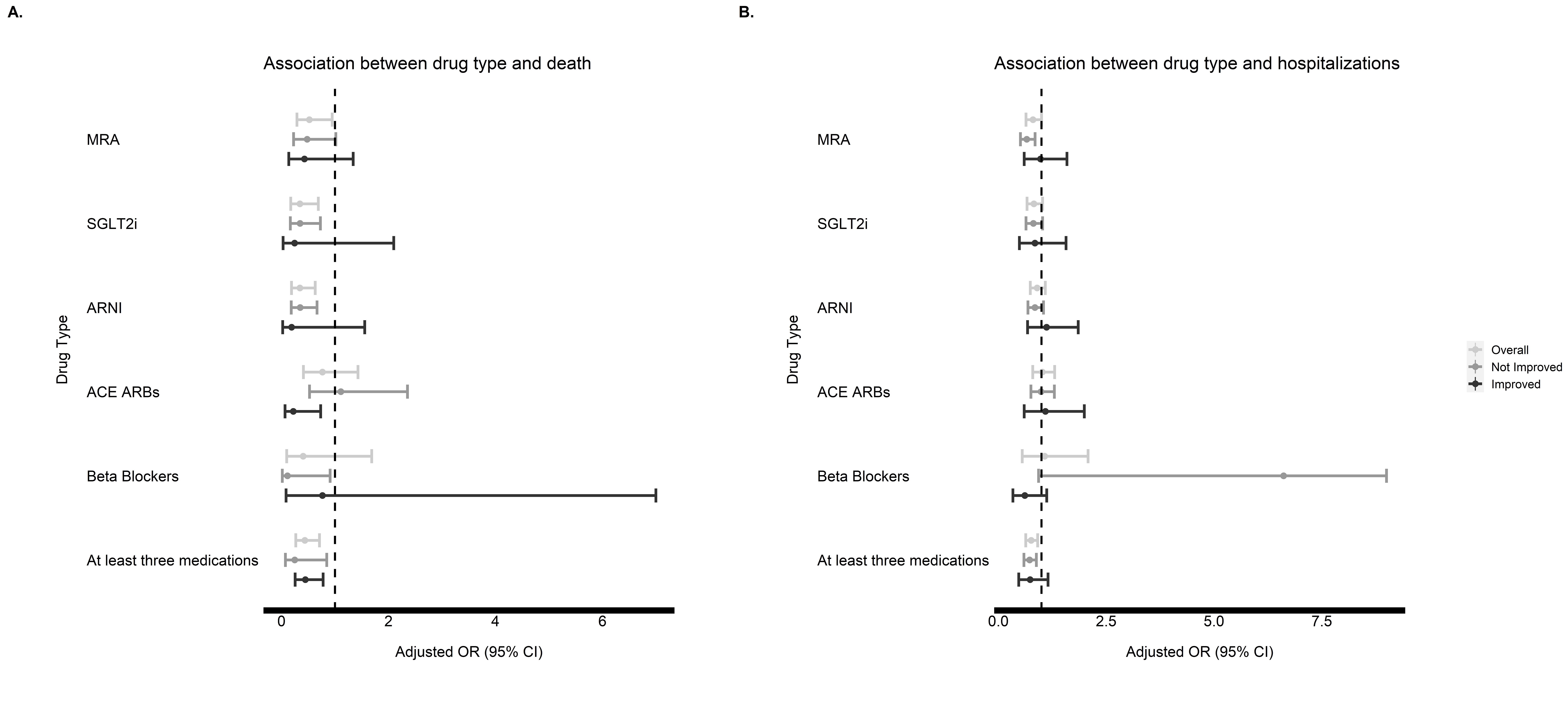

Supplement: Supplementary Figure S3 — Association between drug type to death (A) and number of hospitalizations (B). For each variable the square signifies the adjusted OR and the whiskers signify the 95% confidence interval. The OR for each drug is shown for the overall population (bright gray), the unimproved patients (gray) and the improved patients (black). OR>1 means increased factor for death/hospitalization. [file Image3.jpeg]
